# Biological and Histological Parameters as Predictors of Relapse in Ulcerative Colitis: A Prospective Study

**DOI:** 10.4103/1319-3767.80383

**Published:** 2011

**Authors:** Sheenam Azad, Neena Sood, Ajit Sood

**Affiliations:** Department of Pathology, SGRR Institute of Medical & Health Sciences, Patel Nagar, Dehradun, India; 1 Department of Pathology, DMC & H, Ludhiana, Punjab, India; 2 Department of Gastroenterology, DMC & H, Ludhiana, Punjab, India

**Keywords:** Predictors, remission, relapse, quiescent phase, ulcerative colitis

## Abstract

**Background/Aim:**

Ulcerative colitis is a chronic inflammatory disease of unknown etiology characterized by periods of remission and relapses. This study has been carried out in a group of North Indian patients, where the disease has shown an increasing prevalence and frequent relapses. Hence, there is a need to predict relapse for better management and to reduce morbidity. To assess the importance of biological and histological parameters in predicting relapse when the disease is in quiescent phase.

**Materials and Methods:**

A prospective study of twenty-six patients with quiescent ulcerative colitis was carried out in Dayanand Medical College and Hospital, Punjab. Only patients with clinical and endoscopic remission at the time of screening visit were included. Hemoglobin, erythrocyte sedimentation rate (ESR), C- reactive protein (CRP) and serum Interleukin-6 (IL-6) levels were measured. The baseline colonoscopic mucosal biopsies were retrieved and studied. Follow-up was conducted for one year at monthly interval or earlier if relapse occurred.

**Results:**

Fifteen out of twenty-six patients (57.69%) had evidence of clinical relapse during the follow-up. Hemoglobin, ESR, CRP and IL-6 levels were not found to be significant predictors of relapse. Increased number of eosinophils and neutrophils in the lamina propria were observed to be associated with significantly higher relapse rate.

**Conclusion:**

A higher risk of relapse in patients with quiescent colitis can be predicted by the presence of increased number of eosinophils and neutrophils in the lamina propria.

Ulcerative colitis is a chronic inflammatory disease of unknown etiology characterized by recurring episodes of inflammation primarily limited to the mucosal layer of the colon. It is a worldwide disorder with significant geographical heterogeneity, the highest prevalence rates having been reported from Northwest Europe and North America.[1] The incidence and prevalence of ulcerative colitis in the Indian subcontinent is rising and the disease frequency is not much less than that reported from Europe and North America.[2]

Clinical course of ulcerative colitis is characterized by periods of remission punctuated by clinical exacerbations. Active disease can be associated with a rise in acute phase reactants like C-reactive protein (CRP), erythrocyte sedimentation rate (ESR) and decrease in hemoglobin (Hb).[3] Cytokines play an important regulatory role in ulcerative colitis; there is an increased production of most of the major proinflammatory cytokines [Interleukin-1, Interleukin-6, Interleukin-8 (IL-1, IL-6, IL-8)] and tumor necrosis factor-α (TNF-α).[4,5] These serum parameters may be useful as markers of disease activity.

Various studies have reported one-year relapse rates ranging from 58 to 89% in patients receiving placebo; with medication, yearly relapse rates are reduced to 12-50%.[6] Thus, it is important to identify patients with inactive ulcerative colitis who are likely to relapse subsequently so that an optimal maintenance therapy can be planned. This study has been carried out in a group of patients in North India, where the disease has shown an increasing prevalence, with the aim to assess the importance of biological and histological parameters in predicting relapse when the disease is in quiescent phase.

## MATERIALS AND METHODS

This was a prospective study of 26 patients with quiescent ulcerative colitis who presented to Department of Medicine (Gastroenterology), Dayanand Medical College and Hospital (DMC and H), Ludhiana. The diagnosis of ulcerative colitis was based on the following clinical, endoscopic, and histological criteria: (i) history of diarrhoea with or without blood and/or mucous in the stool; (ii) classical sigmoidoscopy picture seen as diffusely granular, friable, or ulcerated mucosa without rectal sparing or skip lesions; and (iii) histopathological findings suggestive of ulcerative colitis.[2] Only patients in clinical and endoscopic remission at the time of screening visit were included in our study. Clinical remission was defined as baseline bowel function without blood in faeces and absence of steroid therapy. Endoscopic remission was taken as either normal mucosa with clearly visible vascular pattern or erythema with loss of vascular pattern but no bleeding.[6]

ESR was measured by Westergren’s method. C-reactive protein (CRP) was measured quantitatively by turbidimetric immunoassay and serum levels of IL-6 were measured by enzyme amplified sensitivity immunoassay (EASIA).

The mucosal biopsies were retrieved by colonoscope at baseline and were fixed in 10% neutral formalin. These were, then, processed and sections stained with hematoxylin and eosin (H and E) and studied for various histological parameters viz. structural change (crypt distortion), cryptitis, crypt abscess (crypt destruction), chronic inflammatory cells in lamina propria, increased eosinophils and neutrophils in lamina propria and basal lymphoid aggregates. They were graded as per criteria laid down by Geboes *et al*.[7]

**Table:** 

Grade 0		Structural (architectural change)
Subgrades	0.0	No abnormality
	0.1	Mild abnormality
	0.2	Mild or moderate diffuse or multifocal abnormalities
	0.3	Severe diffuse or multifocal abnormalities
Grade I		Chronic inflammatory infiltrate
Subgrades	1.0	No increase
	1.1	Mild but unequivocal increase
	1.2	Moderate increase
	1.3	Marked increase
Grade 2		Presence of eosinophils and neutrophils in the lamina propria
2A Eosinophils	2A.0	No increase
	2A.1	Mild but unequivocal increase
	2A.2	Moderate increase
	2A.3	Marked increase
2B Neutrophils	2B.0	None
	2B.1	Mild but unequivocal increase
	2B.2	Moderate increase
	2B.3	Marked increase
Grade 3		Neutrophils in epithelium
	3.0	None
	3.1	< 5 % crypts involved
	3.2	≤50% crypts involved
	3.3	> 50% crypts involved
Grade 4		Crypt destruction
	4.0	None
	4.1	Probable local excess of neutrophils in part of crypt
	4.2	Probable marked attenuation
	4.3	Unequivocal crypt destruction

To eliminate inter-observer bias, two pathologists independently reviewed all the cases of quiescent colitis. The patients were followed up for one year at monthly interval or earlier if relapse occurred, which was defined as worsening of bowel function associated with rectal bleeding.

The data so collected was subjected to statistical analysis, mean and standard deviation computed and Chi-square test applied to see the association between two variables. Student’s t-test was applied to compare the means of two graphs. The significance of difference was seen at appropriate degree of freedom at 1% and 5% level of significance.

## RESULTS

This prospective study included 26 patients (15 males and 11 females) with quiescent ulcerative colitis. The age of the patients varied from 15 to 65 years (mean 37.61 ± 11.74 years). Majority of the patients, 80.77% (21/26) were in the age group of 15-45 years. The duration of disease varied from 1 year to 20 years (Mean 5.60 ± 4.73 years). History of previous relapses was present in 84.62% (22/26) of patients. The mean number of previous relapses were 2.00 ±1.69 [Table 1]. Of the 26 patients, 57.69% (15/26) had evidence of clinical relapse during the follow-up period. The biological and histological differences among the relapsers and non-relapsers are shown in Tables 2 and 3. Duration of the disease, increased number of previous relapses, hemoglobin, ESR, CRP and IL-6 levels were not found to be significant predictors of relapse.

**Table 1:** Baseline clinical parameters in relapsers and non-relapsers

Parameter	Relapsers (*n* = 15)	Non-relapsers (*n* = 11)	Significance
Mean age (years)	35.33 ± 13.9	40.72 ± 9.07	t-value 0.82^NS^*
Age range (years)	16 - 65	24 - 58	χ^2^ =2.67^NS^*
Mean duration of disease in years	5.37 ± 4.75	5.90 ± 4.92	t-value 0.44^NS^*
Mean number of previous relapses	2.53 ± 1.73	1.40 ± 1.4	t-value 1.74^NS^*

NS* - Not significant

**Table 2:** Mean and range of biological parameters in relapsers and non-relapsers

Biological parameters	Relapsers (*n* = 15)	Non-relapsers (*n* = 11)	Significance
Hemoglobin (g/dl)	13.05 ± 1.54	12.35 ± 1.47	t-value=0.80^NS^*
	(10.8 - 15.4)	(9.8 - 15.0)	
ESR† (mm/h)	10.87 ± 11.51	19.18 ± 24.65	t-value=0.76^NS^*
	(5 - 46)	(4 - 90)	
CRP (mg/l)	3.07 ± 3.43	2.45 ± 2.33	t-value=0.56^NS^*
(Normal 0-6)	(0 - 13)	(0 - 6)	
IL-6 pg/ml	17.78 ± 20.96	10.14 ± 3.73	t-value=0.85^NS^*
(Normal 3-8.5)	(5.5 - 91.8)	(6.1 - 18.3)	

Figures in parenthesis indicate range of the measured parameter in the study group.

NS* - Not significant.

† Normal ESR values in different sexes and age groups were calculated on the basis of the table “ESR ranges in health” given by Lewis.[8]

**Table 3:** Summary of histological findings in relapsers and non-relapsers

Histological parameters	Grade and subgrade	Relapsers (*n* = 15)	Non-relapsers (*n* = 15)	Significance
Structural change (crypt distortion)	Grade 0	15 (100)	9 (81.82)	Not significant
No abnormality	0.0	0	2	
Mild abnormality	0.1	10	7	
Mild or moderate diffuse or multifocal abnormalities	0.2	4	2	
Severe diffuse or multifocal abnormalities	0.3	1	0	
Chronic inflammatory cells	Grade 1	15 (100)	8 (72.73)	Not significant
No increase	1.0	0	3	
Mild but unequivocal increase	1.1	11	8	
Moderate increase	1.2	3	0	
Marked increase	1.3	1	0	
Eosinophils in lamina propria	Grade 2A	11 (73.34)	1 (9.10)	*P*<0.01 significant
No increase	2A0	4	10	
Mild but unequivocal increase	2A1	9	1	
Moderate increase	2A2	1	0	
Marked increase	2A3	1	0	
Neutrophils in lamina propria	Grade 2B	10 (66.67)	1 (9.10)	*P*<0.01 significant
None	2B0	5	10	
Mild but unequivocal increase	2B1	5	1	
Moderate increase	2B2	5	0	
Marked increase	2B3	0	0	
Neutrophils in epithelium (cryptitis)	Grade 3	8 (53.33)	0	Cannot be determined
None	3.0	7	11	
< 5 % crypts involved	3.1	8	0	
≤ 50% crypts involved	3.2	0	0	
> 50% crypts involved	3.3	0	0	
Crypt destruction (crypt abscess)	Grade 4	1 (6.67)	0	Cannot be determined
None	4.0	14	11	
Probable local excess of neutrophils in part of crypt	4.1	0	0	
Probable marked attenuation	4.2	1	0	
Unequivocal crypt destruction	4.3	0	0	
Basal lymphoid aggregates	Present	9 (60)	5 (45.46)	Not significant
	Absent	6	6	

Figures in parenthesis indicate percentage

**Figure 1:**
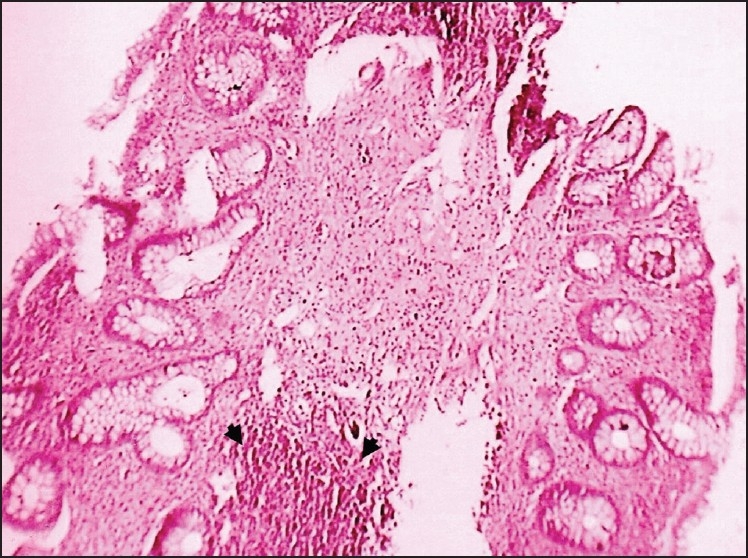
Photomicrograph showing crypt distortion and increase in chronic inflammatory cells in the lamina propria. Arrowheads point toward the basal lymphoid aggregate (H and E, ×100)

**Figure 2:**
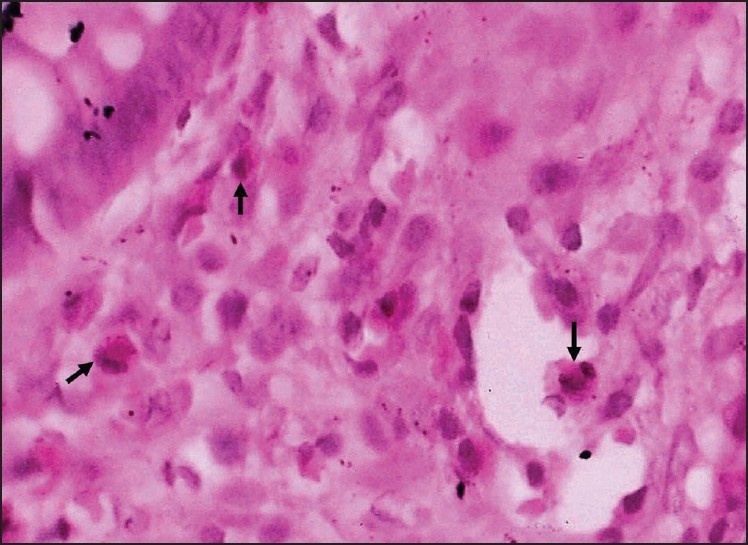
Photomicrograph showing an increase in eosinophils (arrows) in the lamina propria (H and E, ×1000)

**Figure 3:**
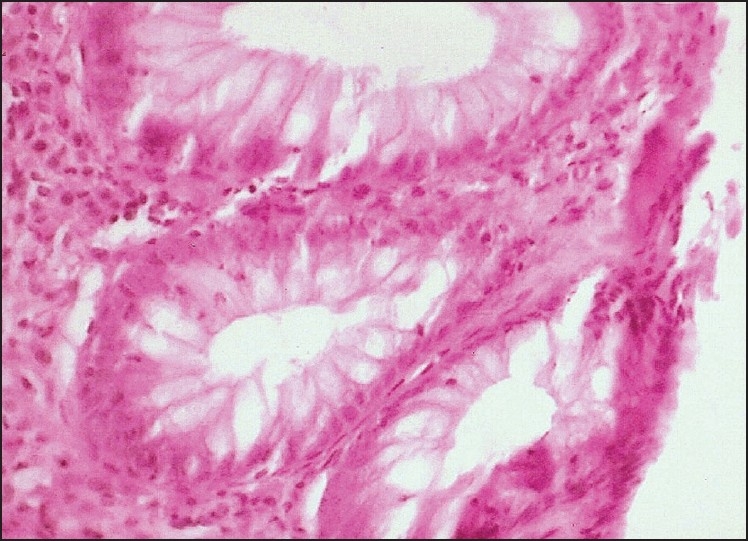
Photomicrograph showing an increase in neutrophils in the lamina propria and epithelium (H and E, ×400)

Among the various histological parameters studied, crypt distortion and increase in chronic inflammatory cells in lamina propria [Figure 1] were noted in all the relapsers and in majority of non-relapsers [Table 3]. Further, increased number of eosinophils and neutrophils in lamina propria [Figures 2 and 3] were seen among relapsers. On the other hand, 90.90% (10 / 11 cases) of the non-relapsers showed no increase in eosinophils and neutrophils in the lamina propria. This difference was found to be statistically significant (*P*<0.01). Cryptitis (presence of neutrophils in the crypt epithelium) was noted in 53.33% (8/15) of the relapsers; however, it was absent in all the non-relapsers. Crypt abscess (presence of neutrophils within the lumen leading to crypt destruction) was seen in only one patient who relapsed early at the beginning of the study. Basal lymphoid aggregates were present in 60 % (9/15cases) of the relapsers and 45.46 % (5/11cases) of the non-relapsers which was not statistically significant.

## DISCUSSION

Ulcerative colitis is a disease characterized by remissions and relapses. The present study has been conducted prospectively in twenty-six patients having quiescent ulcerative colitis with an aim to identify factors that lead to a higher risk of relapse. It is important to recognize such parameters so that patients with an increased risk of relapse can be put on longer maintenance therapy.

The patients in our study group had a relapse rate of 57.69% (15/26), a finding in accordance with other published reports;[9,10] however, Bitton *et al*.[6] have reported a lower relapse rate of 36%. The reason for the higher relapse rate observed in our study group may be either due to differences in the natural course of disease in the Indian subcontinent or poor patient compliance to regular medication. Thus, it is emphasized that due to a higher relapse rate observed in this part of the world, prior identification of various predictors of disease relapse is all the more important.

The patients with quiescent disease who relapsed during the follow up period had a higher frequency of relapses prior to inclusion in this study. Mean number of previous relapses was more among relapsers (2.53±1.73) compared to non-relapsers (1.40 ± 1.4). However, this finding was not statistically significant probably due to smaller sample size. On the other hand, few studies[11,12] have reported that previous relapse frequency has a significant association with the risk of relapse.

Biological markers were assessed as predictors of clinical relapse. Hemoglobin, ESR and CRP were not found to be predictive of clinical recurrence, which is in agreement with other published studies.[6,9] Cytokine IL-6 was studied to assess its role as a marker of disease activity and was found to be increased in 73.33 % (11/15 cases) of the relapsers as well as in 45.45 % (5/11cases) of the non-relapsers which was not statistically significant. It has been reported that IL-6 might be involved in the pathogenesis of ulcerative colitis and serum levels of these cytokines correlate with disease activity.[13,14] In our study, difference between the mean value of IL-6 among relapsers and non-relapsers was not significant (t-value=0.85) and is in concordance with a previous report by Bitton *et al*.[6]

Most patients with ulcerative colitis run a risk of relapse. Even when symptoms improve after treatment for an acute attack, sigmoidoscopic evidence of the inflammatory process often lingers behind.[15] Moreover, the histological evidence of inflammation is commonly seen in patients who achieve completely normal mucosal appearance. In this study, we tried to determine the prognostic importance of microscopic inflammation along with biological parameters. We found that the presence of increased number of eosinophils and neutrophils in the lamina propria were significantly associated with an early relapse. Crypt distortion as well as a mild increase in chronic inflammatory cells in the lamina propria was a common finding among both relapsers and non-relapsers. Cryptitis was seen in 52.33 % (8/15) of the relapsers and was absent in all the non-relapsers. Thus, presence of cryptitis was seen only among patients who relapsed during the course of the study. Similarly, evidence of crypt destruction was seen in only one patient who presented early with clinical relapse within two months of beginning of the study. Probably, this patient was in histological relapse that preceded the clinical relapse.

Histological abnormalities are prevalent in patients with clinically and quiescent colitis. Appreciable microscopic inflammation, especially acute inflammatory cells, were associated with increase in relapse rate.[16,17] Geboes *et al*.[7] found a good correlation between the location of neutrophil and occurrence of crypt destruction. Thus, reduction or disappearance of neutrophils in the epithelium in consecutive biopsies is most likely a sign of reduction of disease activity and could indicate the efficacy of a given treatment.

Similarly, both basal lymphoid hyperplasia (a diffuse increase in lymphocytes at crypt bases) and basal lymphoid aggregates (larger, more discrete nodular aggregates of lymphoid cells at the base of crypts) have been earlier reported as highly discriminant and more common in patients with inflammatory bowel disease.[18,19] In the present study, we found basal lymphoid aggregates in 60% (9/15) of the relapsers and 45% (5/11) of non-relapsers which was not statistically significant. Thus, basal lymphoid aggregates might be a feature of ulcerative colitis but were not of predictive value for disease relapse, a finding similar to that reported by Bitton *et al*.[6]

## CONCLUSION

Among all the biological and histological parameters studied, presence of increased eosinophils and neutrophils in the lamina propria were found to be the most significant predictors of relapse in the present study. In addition, cryptitis was seen only among relapsers. Hence, these patients may be offered more intensive maintenance therapy to prevent future relapse.
